# Therapeutic effect and mechanism of sivelestat sodium on acute lung injury: A randomized controlled trial

**DOI:** 10.1097/MD.0000000000042703

**Published:** 2025-09-26

**Authors:** Yaqing Zhou, Guihua Chen, Jianhua Xu, Haiyan Wang, Qiuxia Ji, Aiming Liu, Zunguo Pu, Xuehua Pu, Ying Wang

**Affiliations:** a Nanjing Medical University, Nanjing, Jiangsu Province, China; b Department of Critical Care Medicine, Affiliated Hai’an Hospital of Nantong University, Hai’an County, Nantong City, Jiangsu Province, China; c Haian Hospital of Traditional Chinese Medicine Affiliated to Nanjing University of Chinese Medicine, Nantong City, Jiangsu Province, China; d Department of Clinical Pharmacy, Affiliated Hai’an Hospital of Nantong University, Hai’an County, Nantong City, Jiangsu Province, China; e Department of Critical Care Medicine, The Affiliated Taizhou People’s Hospital of Nanjing Medical University, Taizhou, Jiangsu Province, China; f Department of Critical Care Medicine, Nantong First People’s Hospital, Nantong City, Jiangsu Province, China.

**Keywords:** acute lung injury, inflammatory factors, lung function, oxygenation index, sivelestat sodium

## Abstract

**Background:**

To observe the therapeutic effect of sivelestat sodium on acute lung injury and explore its mechanism.

**Methods:**

Eighty-six patients with acute lung injury admitted from April 2022 to December 2023 were selected as the research objects. According to the principle of randomized control and single-blindness, they were randomly divided into the observation group (43 cases) and the control group (43 cases). The control group was given conventional symptomatic treatment, while the observation group was treated with sivelestat sodium on this basis. The mechanical ventilation, ICU stay, hospital stay, and 28-day mortality were compared between the 2 groups. The differences in pulmonary function, inflammatory factors, oxygenation index (PaO_2_/FiO_2_), and respiratory rate (RR) between the 2 groups were detected and compared.

**Results:**

The duration of mechanical ventilation, ICU stay, and hospitalization in the observation group were significantly shorter than those in the control group, while there was no difference in 28-day mortality between the 2 groups (*P* > .05). Before treatment, pulmonary function indexes were compared between the 2 groups (*P* > .05). On the 5th and 7th days of treatment, the vital capacity and maximum ventilation volume of the 2 groups were significantly increased compared with those before treatment, and the values of pulmonary function indexes in the observation group at the same time point were better than those in the control group (*P* < .05). Before treatment, the inflammatory factors, PaO_2_/FiO_2_, and RR in the 2 groups were compared (*P* > .05). On the 5th and 7th day after treatment, the tumor necrosis factor-α, interleukin-1β, RR, and intercellular adhesion molecule-1 (ICAM-1) in the 2 groups were significantly decreased compared with those before treatment, while the NOD-like receptors (NLR) family pyrin domain containing 6 (NLRP6) and PaO_2_/FiO_2_ were significantly increased. At the same time, the values of tumor necrosis factor-α, interleukin-1β, ICAM-1, and RR after decreased and NLRP6 and PaO_2_/FiO_2_ after increased in the observation group were better than those in the control group (*P* < .05).

**Conclusion:**

Sivelestat sodium can improve lung function, correct PaO_2_/FiO_2_, and promote rehabilitation in patients with acute lung injury, which may be related to the regulation of NLRP6, ICAM-1, and other inflammatory factors.

## 1. Introduction

Acute lung injury is a serious disease secondary to trauma, infection, poisoning, or burns. Under the influence of various factors, the pulmonary inflammatory response of patients is out of control, resulting in diffuse alveolar and pulmonary interstitial edema and acute hypoxic respiratory insufficiency. Untimely treatment can cause systemic inflammatory response syndrome, multiple organ dysfunction, and other serious consequences, even endanger the lives of patients.^[1]^ At present, the conventional treatment for acute lung injury includes oxygen inhalation, anti-infection, anti-inflammation, nutritional support, maintenance of water electrolyte, and acid–base balance. But the overall effect is not ideal, there are still a considerable number of patients with poor prognosis.^[2]^

Sivelestat sodium is a kind of neutrophil elastase inhibitor, which was born in Japan in the early 1990s. It can inhibit the infiltration of inflammatory cells by selectively inhibiting neutrophil elastase, and reduce the inflammatory injury of lung tissue. At present, it is widely used in the treatment of acute lung injury, acute respiratory distress syndrome, new corona pneumonia and other diseases, and has achieved good results.^[3,4]^ However, its mechanism of action has not been fully elucidated. In this study, we observed the therapeutic effect of sivelestat sodium on acute lung injury and explored its mechanism.

Core tips: sivelestat sodium is a new drug for clinical treatment of acute lung injury. This study found that it can improve lung function, correct oxygenation index (PaO_2_/FiO_2_), and promote rehabilitation, which is consistent with the existing research results. This study also found that the protective effect of sivelestat sodium on lung function may be related to the regulation of NOD-like receptors (NLR) family pyrin domain containing 6 (NLRP6), intercellular adhesion molecule-1 (ICAM-1), and other inflammatory factors.

## 2. Data and methods

### 2.1. General information

**Figure 1.**
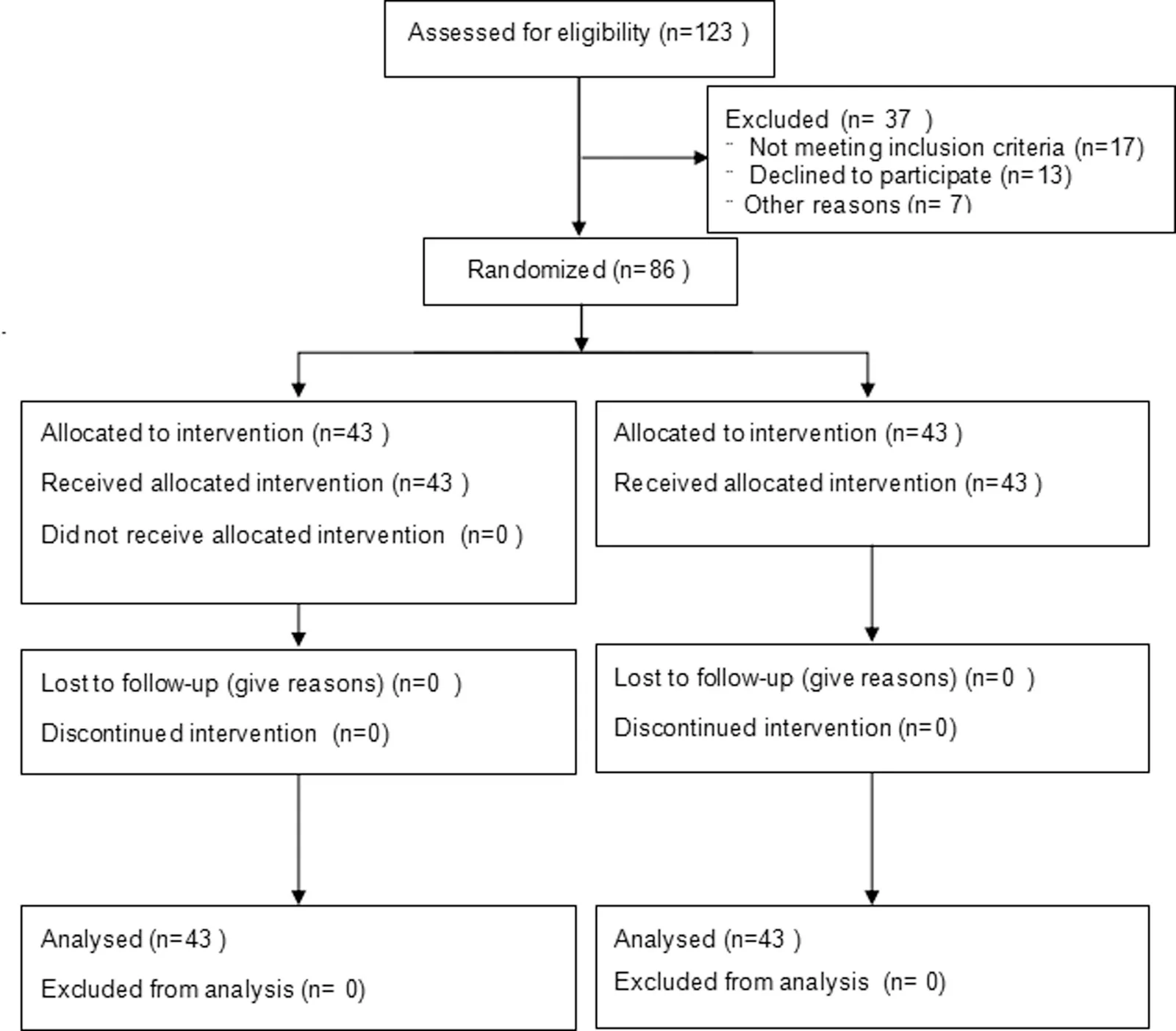
CONSORT flow diagram.

Eighty-six patients with acute lung injury admitted in Hai’an People’s Hospital and Nantong First People’s Hospital from April 1, 2022 to December 31, 2023 were selected as the research objects. The patients were divided into the observation and control groups through single-blinded simple randomization using a random number table and the sealed envelope system. Matching was performed for the 2 groups in terms of general characteristics in accordance with the CONSORT guidelines^[5]^ (Fig. 1). The primary outcome measure was maximum ventilation. The sample size is determined using the sample size estimation formula for comparing the mean of 2 samples^[6]^: $n_{1}=n_{2}=2\times {\left[\frac{\left(t_{\alpha }+t_{\beta }\right)\times s}{\delta }\right]}^{2}$, with a 0.05 margin of error, and 95% confidence level, α = 0.05, β = 0.1, we can obtained from the t value table: t_α_ = 1.96, t_β_ = 1.28,and our s = 0.84, δ = 0.63,therefore $n=2\times {\left[\frac{\left(1.96+1.28\right)\times 0.84}{0.63}\right]}^{2}=37.3$. According to the principle of randomized control and single-blindness, they were randomly divided into the observation group (43 cases) and the control group (43 cases). The data between the 2 groups were balanced. The study was approved by Medical Ethics Committee of Hai’an People’s Hospital (HKL2022106). Obtain written informed consent from the patient or their guardian. The study was registered with the China Clinical Trial Center (registration number: ChiCTR2300075392).

Inclusion criteria: acute lung injury meets the criteria set by the Chinese Medical Association in 2006.^[7]^ Age ≥ 18 years, ≤65 years without gender restriction. Good compliance, with treatment and efficacy evaluation. Exclusion criteria: shock or solid malignant tumor. Upper airway injury. Severe heart, liver, and kidney dysfunction. Anaphylaxis to sivelestat sodium. Pregnancy, pregnancy preparation, lactation. There are blood and immune system diseases.

### 2.2. Method

The control group was given conventional symptomatic treatment, low flow oxygen inhalation, active treatment of primary diseases or trauma, and positive anti-infection, anti-inflammatory, rehydration, parenteral nutrition support. On this basis, the observation group was treated with sivelestat sodium (Shanghai Huilun Jiangsu Pharmaceutical Co., Ltd., specification: 0.1 g, Chinese medicine Zhunzi H20203093). 0.3 g of sivelestat sodium was added to 50 mL of 0.9% sodium chloride injection, continuous intravenous infusion, and the speed was 0.2 mg/(kg·h). Both groups were evaluated after 7 days of treatment.

### 2.3. Observation indicators and detection methods

The mechanical ventilation, ICU stay, hospital stay, and 28-day mortality were compared between the 2 groups. The differences in lung capacity (L), maximum ventilation (L/min), inflammatory factors, PaO_2_/FiO_2_, and respiratory rate (RR) between the 2 groups were detected and compared.

The vital capacity, maximum ventilation indexes of the 2 groups were detected before treatment, 5 days and 7 days after treatment. The detection instrument: Anke FGC-A pulmonary function detector. Before treatment, 5 mL venous blood samples of patients under fasting state were collected on the 5th and 7th days of treatment. The samples were centrifuged at a rotational speed of 3000 r/min and a duration of 10 minutes. Serum samples were collected and detected for tumor necrosis factor-α (TNF-α), interleukin (IL)-1β, ICAM-1, and NLRP6 by enzyme-linked immunosorbent assay kit (Shanghai Enzyme-linked Biotechnology Co., Ltd., Shanghai, China). Detection instrument: Infinit2000 multi-functional microplate reader from Tecan, Switzerland.

### 2.4. Statistical method

The data were processed by SPSS19.0. The K-S method was used to test the normality of the measurement data. The measurement data conforming to the normal distribution were described by (χ±s). The *t* test was used for comparison. The enumeration data were tested by the 4-grid table method or the *χ*^2^ test of row × list, and *P* < .05 was statistically significant.

## 3. Results

### 3.1. Analysis of 2 groups of baseline data

Gender (55.81% vs 51.16%/44.19 vs 48.84, *P* = .665), age (52.85 ± 10.46 vs 51.69 ± 9.54, *P* = .592), body mass index (23.25 ± 1.89 vs 23.19 ± 2.01, *P* = .887), underlying diseases (34.88% vs 39.53%/65.12% vs 60.47%, *P* = .655), smoking history (27.91% vs 32.56%/72.09% vs 67.44%, *P* = .639), PaO_2_/FiO_2_ (221.25 ± 28.65 vs 217.56 ± 31.14, *P* = .569) were compared between the 2 groups (*P* > .05). As shown in Table 1.

**Table 1:** Baseline data analysis of the 2 groups.

Normal information	Control group (n = 43)	Observation group (n = 43)	χ^2^ or *t* value	*P* value
Gender [n (%)]			0.187	.665
Male	24 (55.81)	22 (51.16)		
Female	19 (44.19)	21 (48.84)		
Age [(χ±s), age]	52.85 ± 10.46	51.69 ± 9.54	0.537	.592
BMI [(χ±s), kg/m^2^]	23.25 ± 1.89	23.19 ± 2.01	0.143	.887
PaO₂/FiO₂ [(χ±s), mm Hg]	221.25 ± 28.65	217.56 ± 31.14	0.572	.569
Basic illness [n (%)]			0.199	.655
Lung infection	15 (34.88)	17 (39.53)		
Sepsis	28 (65.12)	26 (60.47)		
Drinking history [n (%)]			0.212	.654
Have	15 (34.88)	13 (30.23)		
None	28 (65.12)	30 (69.77)		
Smoking history [n (%)]			0.221	.639
Have	12 (27.91)	14 (32.56)		
None	31 (72.09)	29 (67.44)		

BMI = body mass index.

### 3.2. Comparison of mechanical ventilation, ICU stay, hospital stay, and 28-day mortality between the 2 groups

The duration of mechanical ventilation (6.99 ± 1.59 vs 5.33 ± 1.54, *P* = .000), ICU stay (8.97 ± 2.58 vs 7.74 ± 1.96, *P* = .015), and hospitalization (18.85 ± 4.15 vs 15.12 ± 2.89, *P* = .000) in the observation group were significantly shorter than those in the control group, while there was no difference in 28-day mortality (6.98% vs 2.33%, *P* = .306) between the 2 groups (*P* > .05) (see Table 2).

**Table 2:** Comparison of mechanical ventilation, ICU stay, length of stay, and 28-day mortality between the 2 groups.

Group	n	Mechanical ventilation time [(χ±s), d]	ICU stay [(χ±s), d]	Hospital stay [(χ±s), d]	28-d case fatality rate [n (%)]
Control group	43	6.99 ± 1.59	8.97 ± 2.58	18.85 ± 4.15	3(6.98)
Observation group	43	5.33 ± 1.54	7.74 ± 1.96	15.12 ± 2.89	1(2.33)
*t* or χ^2^ value		4.918	2.489	4.837	1.049
*P* value		.000	.015	.000	.306

### 3.3. Comparison of pulmonary function indexes between 2 groups

Before treatment, the lung capacity (1.23 ± 0.24 vs 1.25 ± 0.23, *P* = .694), maximum ventilation (31.52 ± 3.98 vs 30.89 ± 4.17, *P* = .476) of the 2 groups were compared (*P* > .05). On the 5th (1.62 ± 0.33 vs 1.25 ± 0.23, *P* = .040), (40.02 ± 4.24 vs 30.89 ± 4.17, *P* = .011) and 7th (1.88 ± 0.41 vs 1.25 ± 0.23, *P* = .044), (46.96 ± 4.19 vs 30.89 ± 4.17, *P* = .000) days of treatment, the vital capacity and maximum ventilation volume of the 2 groups were significantly increased compared with those before treatment, and the values of lung capacity (1.62 ± 0.33 vs 1.48 ± 0.29, *P* = .040), (1.88 ± 0.41 vs 1.71 ± 0.36, *P* = .044), maximum ventilation (40.02 ± 4.24 vs 36.69 ± 4.05, *P* = .011), (46.96 ± 4.19 vs 42.04 ± 4.32, *P* = .000) in the observation group at the same time point were better than those in the control group (*P* < .05) (see Table 3).

**Table 3:** Comparison of pulmonary function indexes between the 2 groups (χ±s).

Group	n	Lung capacity (L)			Maximum ventilation (L/min)		
		Before therapy	Heal 5 d	Heal 7 d	Before therapy	Heal 5 d	Heal 7 d
Control group	43	1.23 ± 0.24	1.48 ± 0.29*	1.71 ± 0.36*	31.52 ± 3.98	36.69 ± 4.05*	42.04 ± 4.32
Observation group	43	1.25 ± 0.23	1.62 ± 0.33*	1.88 ± 0.41*	30.89 ± 4.17	40.02 ± 4.24*	46.96 ± 4.19
*t* value		0.395	2.090	2.043	0.717	3.724	5.361
*P* value		.694	.040	.044	.476	.011	.000

* Compared with the control group, *P* < .05.

### 3.4. Comparison of inflammatory-related factors between the 2 groups

Before treatment, inflammatory factors were compared between the 2 groups (*P* > .05). On the 5th and 7th days of treatment, TNF-α, IL-1β, and ICAM-1 in the 2 groups were significantly decreased compared with those before treatment, while NLRP6 was significantly increased, and the values of TNF-α (58.52 ± 4.96 vs 71.25 ± 6.98, *P* = .000), (26.36 ± 4.47 vs 34.74 ± 5.11, *P* = .000), IL-1β (27.11 ± 4.16 vs 33.25 ± 4.25, *P* = .000), (18.25 ± 3.41 vs 23.68 ± 3.89, *P* = .000), and ICAM-1 (15.21 ± 1.89 vs 17.85 ± 2.03, *P* = .000), (11.36 ± 1.54 vs 13.44 ± 1.85, *P* = .000) decreased and NLRP6 (76.69 ± 11.05 vs 67.02 ± 9.52, *P* = .000), (98.63 ± 12.47 vs 89.11 ± 11.63, *P* = .004) increased in the observation group at the same time point were better than those in the control group (*P* < .05) (see Table 4).

**Table 4:** Comparison of inflammation-related factors between the 2 groups (χ±s).

Group	n	TNF-α (ng/L)			IL-1β (ng/L)		
		Before therapy	Heal 5 d	Heal 7 d	Before therapy	Heal 5 d	Heal 7 d
Control group	43	114.25 ± 12.52	71.25 ± 6.98*	34.74 ± 5.11*	46.36 ± 4.98	33.25 ± 4.25*	23.68 ± 3.89*
Observation group	43	108.85 ± 13.34	58.52 ± 4.96*	26.36 ± 4.47*	45.97 ± 5.21	27.11 ± 4.16*	18.25 ± 3.41*
*t* value		1.936	9.749	8.094	0.355	6.770	6.883
*P* value		.056	.000	.000	.724	.000	.000
Group	n	ICAM-1 (ng/L)			NLRP6 (pg/mL)		
		Before therapy	Heal 5 d	Heal 7 d	Before therapy	Heal 5 d	Heal 7 d
Control group	43	23.78 ± 2.19	17.85 ± 2.03*	13.44 ± 1.85*	47.52 ± 8.55	67.02 ± 9.52*	89.11 ± 11.63*
Observation group	43	23.69 ± 2.27	15.21 ± 1.89*	11.36 ± 1.54*	46.95 ± 8.72	76.69 ± 11.05*	98.63 ± 12.47*
*t* value		0.187	6.242	5.666	0.307	4.348	3.661
*P* value		.852	.000	.000	.760	.000	.004

ICAM-1 = intercellular adhesion molecule-1, IL = interleukin, NLRP6 = NLRP inflammatory body 6, TNF-α = the tumor necrosis factor-α.

* Compared with the control group, *P* < .05.

### 3.5. Comparison of PaO_2_/FiO_2_/RR between 2 groups

Before treatment, PaO_2_/FiO_2_, RR were compared between the 2 groups (*P* > .05). On the 5th (30.11 ± 3.58 vs 23.96 ± 3.41, *P* = .000), (254.12 ± 23.69 vs 268.63 ± 24.11, *P* = .006), and 7th (22.23 ± 2.33 vs 18.52 ± 2.09, *P* = .000), (277.01 ± 28.52 vs 293.63 ± 24.28, *P* = .005) day after treatment, RR decreased significantly in both groups, while PaO_2_/FiO_2_ increased significantly, and the values of RR decreased and PaO_2_/FiO_2_ increased in the observation group were better than those in the control group (*P* < .05) (see Table 5).

**Table 5:** Comparison of PaO₂/FiO₂ and RR between the 2 groups (χ±s).

Group	n	PaO₂/FiO₂ (mm Hg)			RR (times/min)		
		Before therapy	Heal 5 d	Heal 7 d	Before therapy	Heal 5 d	Heal 7 d
Control group	43	221.25 ± 28.65	254.12 ± 23.69*	277.01 ± 28.52*	38.96 ± 4.11	30.11 ± 3.58*	22.23 ± 2.33*
Observation group	43	217.56 ± 31.14	268.63 ± 24.11*	293.63 ± 24.28*	39.05 ± 4.06	23.96 ± 3.41*	18.52 ± 2.09*
*t* value		0.572	2.815	2.910	0.102	8.157	7.773
*P* value		.569	.006	.005	.919	.000	.000

PaO_2_/FiO_2_ = oxygenation index, RR = respiratory rate.

* Compared with the control group, *P* < .05.

### 3.6. Comparison of adverse reactions between the 2 groups

There was 1 case of dyspnea in the observation group, and no other adverse reactions such as liver injury, kidney injury, and rash occurred. No adverse reactions occurred in the control group.

## 4. Discussion

Acute lung injury is a common clinical critical disease characterized by noncardiac pulmonary edema and refractory hypoxemia. Studies suggest that lung is the most vulnerable target organ in the development of multiple organ dysfunction syndrome.^[8]^ Stimulated by stressors such as trauma and infection, a large number of neutrophils and mononuclear–macrophages aggregate and activate in the lung, releasing a variety of inflammatory cytokines and oxygen free radicals, causing pulmonary capillary dilation, increased permeability and decreased lung compliance.^[9,10]^ At present, there are many clinical studies on the pathogenesis and treatment of acute lung injury. Some scholars have found that the proportion of Th22 cells and Th17 cells in peripheral blood of sepsis patients with acute lung injury is unbalanced, and the disorder of cytokines is unfavorable to the prognosis of patients.^[11,12]^ Clinically, it has been committed to finding a new safe and effective combination therapy to reduce lung injury and promote disease outcome.

Sivelestat sodium is a neutrophil elastase inhibitor developed by Japanese scientists. It was approved for the treatment of new coronavirus pneumonia in China in 2020, and has good curative effect in reducing potential complications of new coronavirus pneumonia.^[13]^ Some scholars have used sivelestat sodium to treat systemic inflammatory response syndrome and acute lung injury caused by acute pancreatitis, and found that it can better reduce the degree of lung injury and is beneficial to prognosis.^[14]^ In the study, we applied sivelestat sodium to the treatment of acute lung injury, and found that the duration of mechanical ventilation, ICU stay and hospitalization of patients were significantly shorter than those of patients with conventional treatment, while there was no significant difference in the 28-day mortality between the 2 groups. This result suggests that sivelestat sodium in the treatment of acute lung injury can better promote rehabilitation, which is consistent with existing research conclusions. This is due to lung inflammation, neutrophils release a large number of elastase into the lung interstitial, causing lung tissue inflammatory injury.^[15]^ The inhibitory effect of sivelestat sodium on elastase is highly specific, which can reduce the increase of pulmonary vascular permeability and tracheal contraction caused by the activation of elastase, and then inhibit the release of pro-inflammatory factors and apoptosis of lung cells.^[16,17]^ The 28-day mortality rate is related to multiple factors such as primary diseases or trauma, underlying diseases, and treatment measures. Sivelestat sodium has no obvious advantage in reducing mortality.

The pulmonary function and oxygenation index of the 2 groups before and after treatment were also detected. The results showed that the vital capacity, maximal ventilation volume and PaO_2_/FiO_2_ ratio of the patients treated with sivelestat sodium were higher than those of the patients treated with conventional therapy, and RR was lower than that of the patients treated with conventional therapy. The results suggest that sivelestat sodium can improve lung function and correct oxygenation index in patients with acute lung injury. This is because sivelestat sodium can reduce lung tissue inflammatory injury and pulmonary edema, improve lung compliance, reduce lung ischemia–reperfusion injury. After the reduction of pulmonary congestion and edema, the state of excessive ventilation was alleviated, and the RR gradually slowed down. After the improvement of pulmonary ventilation function, the body’ s hypoxic state was corrected.^[18]^

Inflammatory factors play an important role in promoting the occurrence of acute lung injury. TNF-α belongs to multi-effect pro-inflammatory factor, which can increase the release of other pro-inflammatory factors and cause or aggravate inflammatory injury of lung tissue.^[19]^ IL-1β has a strong pro-inflammatory effect, can induce a variety of inflammatory cytokines, chemokines, causing extensive validation time.^[20]^ ICAM-1 can promote the aggregation of inflammatory cells in the lung, damage pulmonary vascular epithelial cells and endothelial cells, and participate in the pathological process of acute lung injury.^[21]^ NLRP6 is a member of the NLRs family that is highly expressed in the intestine and inhibits inflammatory response.^[22]^ In the lipopolysaccharide-induced myocardial injury model in rats, sivelestat sodium reduces the expression of inflammatory factors, such as IL-1β and IL-6, which can act on the ERK1/2 signaling pathway and exert its cardiomyocyte protective effects.^[23]^ In an acute kidney injury model, sivelestat sodium reduced the mRNA expression levels of IL-6, macrophage inflammatory protein-2, monocyte chemotactic protein-1, and tumor necrosis factor-α in the kidneys, confirming that sivelestat sodium may be effective in alleviating renal dysfunction induced by acute kidney injury by inhibiting the TLR4/Myd88/NF-κB pathway.^[24]^ In this study, the levels of serum inflammatory factors were detected, and it was found that the serum TNF-α, IL-1β, and ICAM-1 levels of patients treated with sivelestat sodium for 5 days and 7 days were lower than those of patients treated with conventional therapy, and NLRP6 was higher than that of patients treated with conventional therapy. These results suggest that the therapeutic effect of sivelestat sodium on acute lung injury may be related to the regulation of NLRP6, ICAM-1, and other inflammatory factors.

In conclusion, sivelestat sodium can improve lung function, correct oxygenation index, and promote rehabilitation in patients with acute lung injury, which may be related to the regulation of NLRP6, ICAM-1, and other inflammatory factors.

## Author contributions

**Conceptualization:** Yaqing Zhou, Jianhua Xu, Xuehua Pu, Ying Wang.

**Data curation:** Qiuxia Ji.

**Formal analysis:** Aiming Liu.

**Investigation:** Yaqing Zhou, Zunguo Pu, Ying Wang.

**Methodology:** Yaqing Zhou, Jianhua Xu, Haiyan Wang.

**Resources:** Qiuxia Ji, Zunguo Pu, Ying Wang.

**Supervision:** Haiyan Wang, Xuehua Pu.

**Visualization:** Haiyan Wang, Xuehua Pu.

**Writing – original draft:** Guihua Chen, Jianhua Xu, Aiming Liu.

**Writing – review & editing:** Yaqing Zhou, Guihua Chen, Xuehua Pu.
